# Sonodynamic therapy suppresses matrix collagen degradation in vulnerable atherosclerotic plaque by modulating caspase 3 - PEDF/HIF-1α - MMP-2/MMP-9 signaling in macrophages

**DOI:** 10.1371/journal.pone.0279191

**Published:** 2022-12-27

**Authors:** Yanfeng Tian, Siqi Sheng, Weiwei Gao, Jianting Yao, Ye Tian

**Affiliations:** 1 Department of Cardiology, The First Affiliated Hospital, Cardiovascular Institute, Harbin Medical University, Harbin, China; 2 The Second Affiliated Hospital of Southern University of Science and Technology (The Third People’s Hospital of Shenzhen), Southern University of Science and Technology, Shenzhen, China; Southern University and A&M College, UNITED STATES

## Abstract

**Background:**

The rupture of vulnerable atherosclerotic plaque is the main cause of acute ischemic vascular events, and is characterized by pathological degradation of matrix collagen in the fibrous cap. In a previous study, we reported that 5-aminolevulinic acid-mediated sonodynamic therapy suppressed collagen degradation in rabbit plaque. However, the underlying molecular mechanism has yet to be fully elucidated.

**Methods:**

We applied sinoporphyrin sodium-mediated sonodynamic therapy (DVDMS-SDT) to balloon-denuded rabbit and apolipoprotein E-deficient (ApoE−/−) mouse models to observe collagen content in plaque. Cultured human THP-1 and mouse peritoneal macrophage-derived foam cells were used for *in vitro* mechanistic studies.

**Results:**

We observed that DVDMS-SDT decreased plaque area and increased the percentages of collagen and smooth muscle cells and reduced the percentage of macrophages in rabbit and ApoE-/- mouse advanced plaques. *In vitro*, DVDMS-SDT modulated the caspase 3-pigment epithelium-derived factor/hypoxia-inducible factor-1α (PEDF/HIF-1α)-matrix metalloprotease-2/9 (MMP-2/MMP-9) signaling in macrophage foam cells.

**Conclusions:**

Our findings show that DVDMS-SDT effectively inhibits matrix collagen degradation in advanced atherosclerotic plaque by modulating caspase 3-PEDF/HIF-1α-MMP-2/MMP-9 signaling in macrophage foam cells and therefore represents a suitable and promising clinical regimen to stabilize vulnerable plaques.

## 1. Introduction

The rupture of atherosclerotic plaque is the major cause of ischemic vascular diseases such as acute myocardial infarction, ischemic stroke, and peripheral arterial occlusion [1, 2]. Unstable plaques are characterized by increased concentrations of lipids and macrophages as well as thinner fibrous caps, whose maintenance reflects the production and degradation of the extracellular matrix (ECM) [3]. Collagen is mainly synthesized by smooth muscle cells (SMCs) and degraded by active matrix metalloproteinases (MMPs) produced by macrophages. As the principal component of ECM, collagen provides tensile strength for the fibrous cap to stabilize atherosclerotic plaque [4]. During plaque progression, macrophages, which exaggeratedly accumulate in the lesion overtime, produce a large amount of active MMPs that excessively degrade collagen. The initiation of this process thus reduces the tensile strength of the fibrous cap and promotes plaque destabilization [5]. Therefore, strategies targeting restoration of the balance between the synthesis and degradation of collagen are likely to reinforce plaque stability and increase the survival rate of patients suffering from ischemic vascular diseases.

Regarding therapeutic intervention of MMPs activities, some studies have reported that statins can moderately reduce MMP expression to inhibit collagen degradation in advanced atherosclerotic plaque [6, 7], albeit with several non-negligible systemic side effects [8, 9]. Recently, rosiglitazone, an insulin sensitizer, has been found to stabilize atherosclerotic plaques by modulating collagen deposition and metabolism in the plaques of fat-fed apolipoprotein E-deficient (ApoE-/-) mice [10]. However, increasing evidence of adverse effects of rosiglitazone on the cardiovascular system, including increased incidence of myocardial infarction and cardiovascular-related mortality, has limited its clinical application [11]. Therefore, a site-specific intervention modulating collagen deposition may present a safe and feasible alternative to stabilize atherosclerotic plaque.

Sonodynamic therapy (SDT) is a non-traumatic technique based on the use of low-intensity ultrasound to locally activate a pre-loaded sonosensitizer to generate a controlled amount of reactive oxygen species (ROS), which enables modulation of the cell biology [12–14]. We have previously shown 5-aminolevulinic acid-mediated sonodynamic therapy (ALA-SDT) rapidly stabilized vulnerable plaque by stimulating macrophage apoptosis and suppressing collagen degradation without displaying any remarkable off-target effects [15, 16]. Additionally, we applied a novel sonosensitizer, sinoporphyrin sodium (DVDMS), which was shown to have stronger singlet oxygen generation efficiency compared to PpIX [17, 18]. In the present study, we aimed to further address the underlying intracellular mechanisms of the remarkable therapeutic phenotype. Therefore, we used rabbit and ApoE-/- mouse models of advanced atherosclerosis *in vivo* and THP-1-derived and mouse peritoneal macrophage (MPM)-derived foam cells *in vitro* to exhaustively determine the efficacy of DVDMS-SDT in inhibiting plaque matrix collagen degradation and explore the associated signaling pathways.

## 2. Materials and methods

### 2.1. Advanced atherosclerotic plaque animal models

Adult male New Zealand white rabbits weighing 2.5–3 kg (Solarbio Bioscience & Technology Co., Ltd, Shanghai, China) were fed a 1.5% high cholesterol diet (Solarbio Bioscience & Technology Co., Ltd, Shanghai, China) for 1 week prior to balloon denudation of the right femoral artery, followed by the same diet for an additional 12 weeks. In order to alleviate suffering, the rabbits were sedated with intravenous (i.v.) injection of 3% pentobarbital (30 mg/kg) and intramuscular (i.m.) injection of ketamine (10 mg/kg), xylazine (5 mg/kg), and acepromazine (0.75 mg/kg). The ApoE-/- mice were sedated with intravenous (i.v.) injection of 1% pentobarbital (30 mg/kg) and intramuscular (i.m.) injection of ketamine (10 mg/kg), xylazine (5 mg/kg), and acepromazine (0.75 mg/kg). Anaesthesia was maintained with 1% isoflurane delivered in oxygen. After anaesthesia, balloon injury of the rabbit femoral artery was performed as previously described [18]. Male ApoE-/- mice (aged 6–8 weeks) (Vital River Laboratory Animal Technology Co., Ltd, Beijing, China) were fed a 1% cholesterol diet (Solarbio Bioscience & Technology Co., Ltd, Shanghai, China) for 24 weeks to develop advanced atherosclerotic plaques. All animals were housed in the Animal Care Facilities of Harbin Medical University. Under our intensive care, no animals died during the experiment. The animal protocol was approved by the Human Ethics Committee at Harbin Medical University (IACUC number: 2021095).

### 2.2. DVDMS-SDT procedure for rabbits and ApoE-/- mice

To assess the effects of DVDMS-SDT on atherosclerotic plaque area and composition, 28 rabbits were randomly assigned to four groups: control, DVDMS, ultrasound, and DVDMS-SDT (n = 7). For 36 ApoE-/- mice with atherosclerotic lesions, a group of mice were sacrificed by 3% sodium pentobarbital (160 mg/kg i.p.) and defined as baseline, and the rest were randomly assigned to the control and DVDMS-SDT groups (n = 12). DVDMS-SDT was performed as previously described [18]. After the rabbits and mice were fed a cholesterol diet for 13 and 24 weeks, respectively, the animals were anesthetized at 4 h after DVDMS (4 mg/kg) injection and received ultrasonic sonication (for rabbits: 1.5 W/cm^2^, duty factor 30%; for mice: 0.8 W/cm2, duty factor 30%) for 15 min. Following treatment, the animals in the indicated groups were fed a normal diet containing 0.05% cholesterol and euthanized (3% sodium pentobarbital, 160 mg/kg i.p.) at 1 month post-treatment.

### 2.3. Tissue preparation and histological assay

The rabbits and mice were euthanized with an overdose of 3% sodium pentobarbital (160 mg/kg i.p.). The rabbit right femoral arteries and mouse hearts containing advanced plaques were prepared as previously described [18]. Hematoxylin and eosin (H&E) staining was performed for plaque and lumen areas in rabbits as well as for plaque area in mice. Masson’s trichrome staining was performed to determine the collagen content. Immunohistochemical staining was performed to detect macrophages (for rabbits: anti-RAM-11, 1:1200, Dako, Cat#M0633; for mice: anti-CD68, 1:50, Abcam, Cat#ab955), SMCs (for rabbits: anti-α-actin, 1:2000, Sigma, Cat#A2547; for mice: anti-α-actin, 1:500, Abcam, Cat#ab5694), MMP-2 (for rabbits: anti-MMP-2, 1:400, Bioss, Cat#bs-4605R; for mice: anti-MMP-2, 1:50, Abcam, Cat#ab37150), and MMP-9 (for rabbits: anti-MMP-9, 1:400, Bioss, Cat#bs-4593R; for mice: anti-MMP-9, 1:50, Abcam, Cat#ab38898). Images of the stained sections were obtained using an Olympus IX70 microscope (Olympus, Tokyo, Japan). The amount of collagen, macrophages, SMCs, MMP-2, and MMP-9 were measured using computer-assisted color image analysis software (Image-Pro Plus, version 6.0, Media Cybernetics, Inc., Silver Spring, MA, USA).

### 2.4. Oil Red O staining

Oil Red O staining was performed as described previously [16]. Briefly, the artery segments from the ascending to the thoracic aorta of the ApoE-/- mice were stained with Oil Red O. Images were taken using a Digital Single Lens Reflex camera (Canon, Japan) and the percentage of the lesion area was quantified using IPP software.

### 2.5. Immunofluorescence assays

Mouse hearts were embedded in OCT and serially sectioned at 7-μm thickness. The frozen sections were fixed with 4% paraformaldehyde, blocked with 5% goat serum blocking buffer for 20 min, and washed 3 times with phosphate buffered saline (PBS). Subsequently, the sections were stained with anti-CD68 (1:200, Abcam, Cat#ab955) antibody. The sections were further stained for cleaved caspase 3 (1:200, CST, Cat#9664), PEDF (1:200, Bioss, Cat#bs-20783R), and HIF-1α (1:200, Bioss, Cat#bs-0737R) antibodies. After overnight incubation at 4°C, the sections were washed 3 times with PBS and incubated with FITC-conjugated anti-mouse IgG or Cy3-conjugated anti-rabbit IgG for 1 h at room temperature. The nuclei were counterstained with DAPI and the sections were visualized using a fluorescence microscope. The CD68-positive cells that co-localized with cleaved caspase 3, PEDF or HIF-1α were counted as being positive. The cleaved caspase 3-positive, PEDF-positive, or HIF-1α-positive macrophages per mm^2^ of lesion area were expressed as the number of cells positive for CD68, DAPI, and cleaved caspase 3, PEDF, or HIF-1α divided by plaque area.

### 2.6. Cell culture

Human THP-1- and MPM-derived foam cells were obtained as described previously [18]. The cells were cultured in a humidified atmosphere with 5% CO_2_ at 37°. The ROS scavenger N-acetyl-L-cysteine (NAC) (10 mM, Sigma-Aldrich, Cat#1009005), pan-caspase inhibitor Z-VAD-FMK (10 mM, Selleck-chem, Houston, TX, USA; Cat#S7023), and caspase 3 specific inhibitor Ac-DEVD-CHO (20 μM, Beyotime Biotechnology, Shanghai, China; Cat#C-1206) were cultured with the foam cells for 1 h prior to DVDMS-SDT treatment.

### 2.7. DVDMS-SDT procedure for foam cells

DVDMS was added to the foam cells as described previously [18] followed by ultrasonic sonication for 5 min. To analyze the correlations among caspase 3, PEDF, and HIF-1α, THP-1- and MPM-derived foam cells were lysed for western blot at 0–8 h after DVDMS-SDT treatment.

### 2.8. Enzyme-linked immunosorbent assay

Enzyme-linked immunosorbent assay (ELISA) of the supernatant from THP-1 macrophage foam cells was performed to detect MMP-1, MMP-2, MMP-3, MMP-7, MMP-8, MMP-9, and MMP-10 according to the manufacturer’s instructions. Additionally, ELISA was performed for the supernatant from MPM-derived foam cells to detect MMP-2 and MMP-9 according to the manufacturer’s instructions.

### 2.9. Macrophage foam cells apoptosis assay

Apoptosis of THP-1- or MPM-derived foam cells was detected using Annexin V:FITC Apoptosis Detection Kits (BD Pharmingen). NAC was added to THP-1 or MPMs-derived foam cells 1 h before DVDMS-SDT treatment. Six hours after DVDMS-SDT treatment, the apoptotic rate of cells in each sample were detected as described previously [19].

### 2.10. Protein extraction and western blotting

Whole cell lysates were obtained as described previously [19]. For western blotting, equal amounts of proteins (40–100 μg) were subjected to SDS-PAGE. Nitrocellulose or PVDF membranes containing the transferred proteins were blocked with 5% nonfat dry milk (with 0.1% Tween 20 in Tris-buffered saline) and then incubated with the following primary antibodies overnight at 4°: caspase 9 (1:1000, CST, Cat#9508), cleaved caspase 9 (1:1000, CST, Cat#9505 and Cat#9509), caspase 3 (1:1000, CST, Cat#9662), cleaved caspase 3 (1:1000, CST, Cat#9664), PEDF (1:1000, Abcam, Cat#ab157207), HIF-1α (1:200, Bioss, Cat#bs-0737R), MMP-2 (1:1000, Abcam, Cat#ab37150), MMP-9 (1:1000, Abcam, Cat#ab38898). Actin (1:1000, CST, Cat#4967) was used as a loading control for the whole cell lysates. Horse radish peroxidase-conjugated IgG secondary antibodies (1:4000) were utilized. The protein bands were visualized using a Bio-Rad ChemiDocTMMP Imaging System (UniversalHood III, Bio-Rad Laboratories, Inc., Hercules, CA, USA) using BeyoECL Plus according to the manufacturer’s instructions. All protein bands were quantified using the Quantity One software (Bio-Rad Laboratories) and normalized to actin levels.

### 2.11. Caspase 3 small interfering RNA transfection

Human-specific caspase 3 siRNA and scrambled siRNA were purchased from GenePharma Co., Ltd (Shanghai, China). The siRNA was transfected into THP-1-derived foam cells as described previously [18].

### 2.12. RNA isolation and quantitative real-time PCR

The RNA isolation of the macrophage foam cells was performed as described previously [18]. The primers used in THP-1-derived foam cells were as follows: β-actin forward TGACGGGGTCACCCACACTGTGCCCATCTA, reverse CTAGAAGCATTTGCGGTCGACGATGGAGGG, PEDF forward TTACGCTATGGCTTGGATTCAG, reverse GTCTTCAGTTCTCGGTCTATGTC, HIF-1α forward GCACAGGCCACATTCACG, reverse TTCACAAATCAGCAC-CAAGC, MMP-2 forward TCTACTCAGCCAGCACCCTGGA, reverse TGCAGGTCCACGACGGCATCCA, MMP-9 for-ward TTCGACGTGAAGGCGCAGATGGT, reverse TAGGTCACGTAGCCCACTTGGTC.

### 2.13. Statistical analysis

All quantitative data are expressed as mean ± standard deviation (SD). Statistical analysis was performed using Graphpad 6.0 (GraphPad Software, Inc., La Jolla, CA, USA). The Shapiro–Wilk test was used to determine whether the data were normally distributed. If data were normally distributed, the student’s unpaired t test was used to determine the significant difference between two groups. One-way analysis of variance followed by Dunnett’s or Tukey ’s post hoc testing were used to determine the significant difference between multi-groups. P < 0.05 was considered statistically significant.

## 3. Results

### 3.1. DVDMS-SDT modifies the composition of and stabilizes advanced atherosclerotic plaques in rabbit and ApoE-/- mice

**Fig 1:**
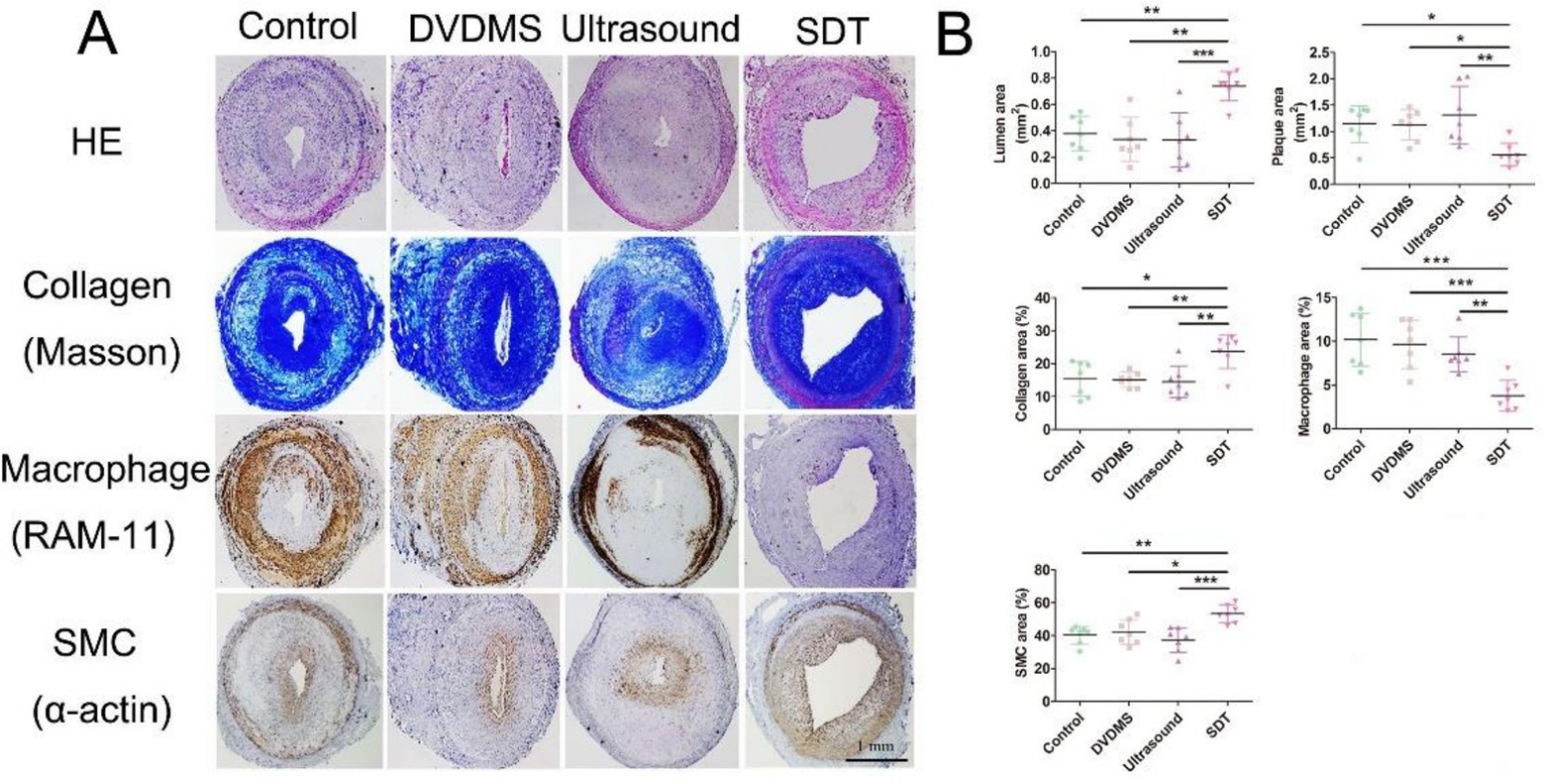
Sinoporphyrin sodium-mediated sonodynamic therapy (DVDMS-SDT) modifies the composition of and stabilizes rabbit femoral advanced atherosclerotic plaque at 1 month after treatment. **(A)** Representative histopathological staining of consecutive plaque sections and **(B)** quantification (n = 7). * P < 0.05, ** P < 0.01, *** P < 0.001.

As reported in our previous study [18], DVDMS accumulated exclusively in macrophages in rabbit and mouse advanced plaques at 4 h after injection rather than in SMCs and endothelial cells. Four hours following the injection of 4 mg/kg DVDMS, ultrasound therapy was performed on rabbit femoral plaques. One month after DVDMS-SDT treatment, the lumen of rabbits and mice subjected to treatment significantly increased by 95.2% (0.38 ± 0.13 vs. 0.74 ± 0.11 mm^2^) and the plaque area significantly decreased by 51% (1.14 ± 0.35 vs. 0.56 ± 0.22 mm^2^) compared with the control group. Additionally, compared with the control group, DVDMS-SDT substantially increased the percentage of collagen by 54.2% (15.38 ± 5.29 vs. 23.71 ± 5.14%) and reduced the percentage of macrophages by 49.7% (10.18 ± 3.03 vs. 3.80 ± 1.73%). Although SMC number was not altered by DVDMS-SDT, the percentage of SMCs was increased by 32.1% (40.36 ± 5.32 vs. 53.31 ± 5.53%) due to the reduction of the plaque size (Fig 1 and S1A Fig in S1 File).

**Fig 2:**
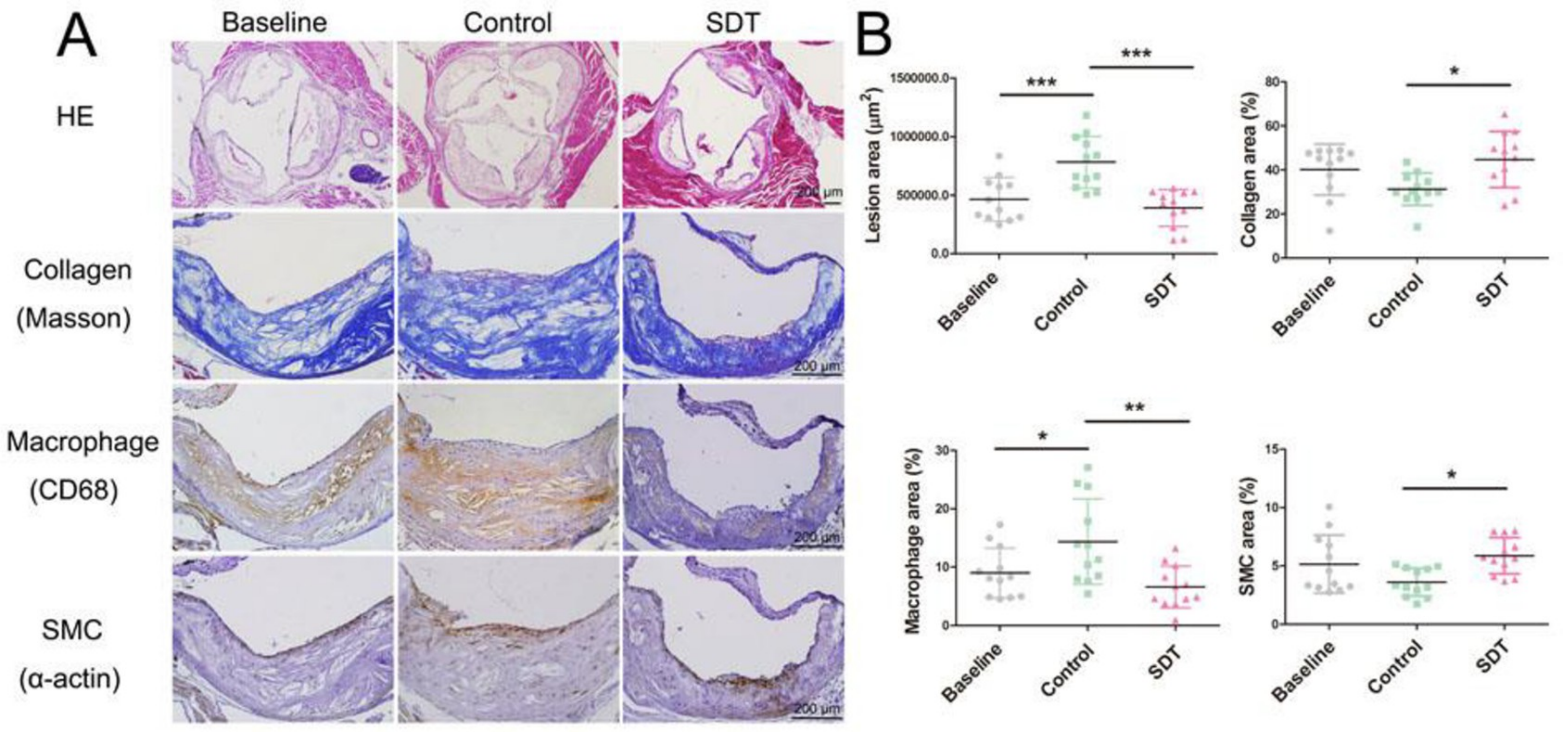
Sinoporphyrin sodium-mediated sonodynamic therapy (DVDMS-SDT) modifies the composition of and stabilizes ApoE-/- mouse advanced atherosclerotic plaque at 1 month after treatment. **(A)** Representative histopathological staining of consecutive plaque sections and **(B)** quantification (n = 12). * P < 0.05, ** P < 0.01, *** P < 0.001.

One month after DVDMS-SDT treatment in ApoE-/- mice, the plaque size and en face lesion area decreased significantly by 50% (781295 ± 220508 vs. 390457 ± 157760 μm^2^) and 42.8% (23.71 ± 5.99 vs. 13.57 ± 3.43%), respectively, compared with the control group. Simultaneously, the percentage of macrophages was drastically reduced by 54.1% (14.36 ± 7.33 vs. 6.59 ± 3.59%) while the percentage of collagen and SMCs increased by 42.8% (31.29 ± 7.33 vs. 44.69 ± 12.75%) and 63.1% (3.61 ± 1.19 vs. 5.88 ± 1.56%), respectively (Fig 2 and S2 Fig in S1 File). Consistent with the data observed in rabbits, the SMCs in the ApoE-/- mice plaque were not altered after DVDMS-SDT treatment (S1B Fig in S1 File).

### 3.2. DVDMS-SDT reduces the expression and secretion of MMP-2 and MMP-9 in macrophage-derived foam cells

**Fig 3:**
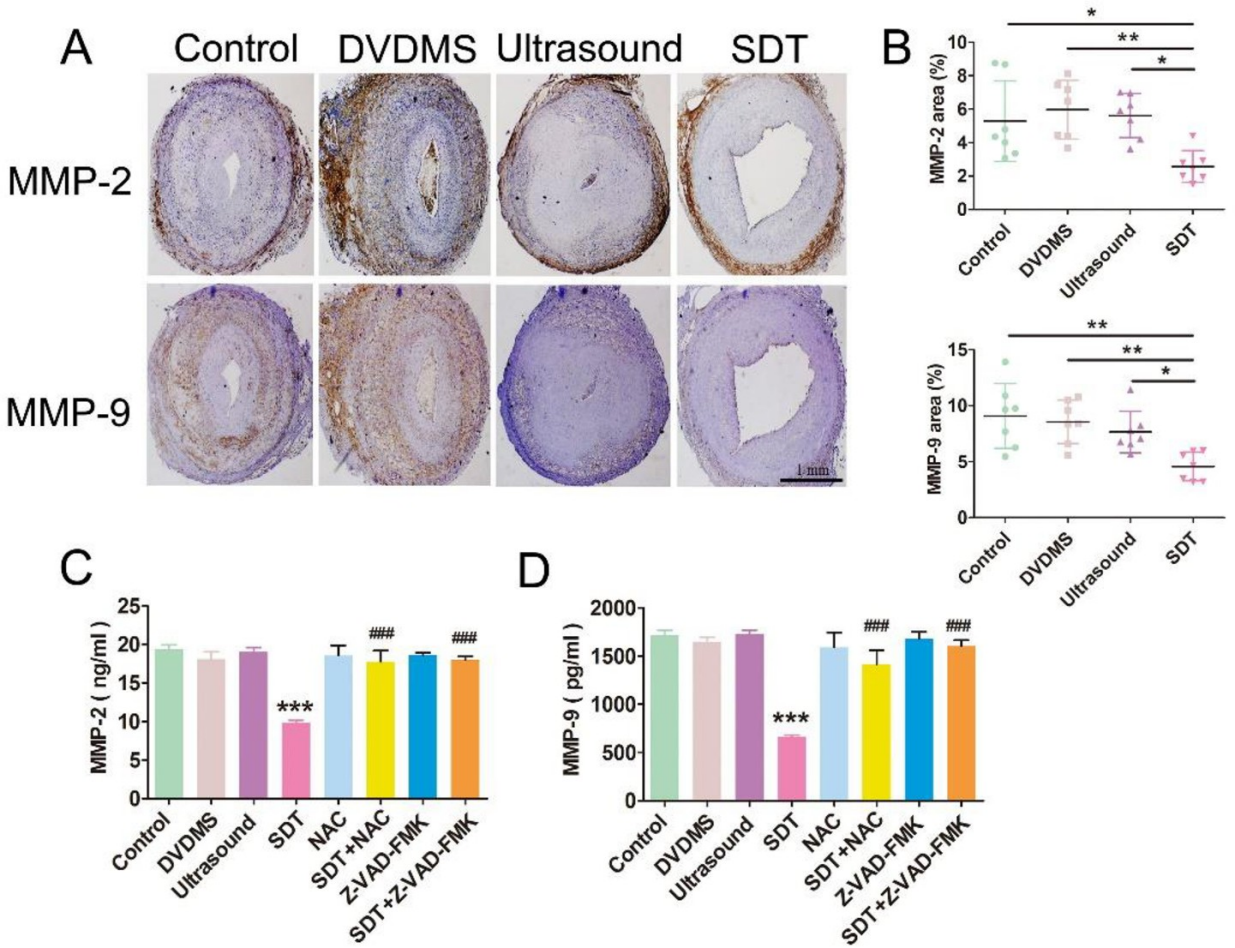
Sinoporphyrin sodium-mediated sonodynamic therapy (DVDMS-SDT) reduces the production of MMP-2 and MMP-9 in rabbit atherosclerotic plaque macrophages. DVDMS-SDT reduced the expression of MMP-2 and MMP-9 in the rabbit plaque at 1 month after treatment. **(A)** Representative histopathological staining of consecutive plaque sections and **(B)** represents quantification (n = 7). THP-1-derived foam cells were pre-treated with N-acetyl-L-cysteine (NAC) or Z-VAD-FMK for 1 h, followed by DVDMS-SDT treatment. At 6 h after treatment, the supernatants in the indicated groups were collected for determination of the concentration of MMP-2 **(C)** and MMP-9 **(D)** by enzyme-linked immunosorbent assay (ELISA). Data on graph were representative of three independent experiments (n = 8). NAC = N-acetyl- L-cysteine. Z-VAD-FMK = methyl (3S)-5-fluoro-3-[[(2S)-2-[[(2S)-3-methyl2 (phenylmethoxycarbonlam ino)butanoyl] ami-no]propanoyl] amino]-4-oxopentanoate. * P < 0.05, ** P < 0.01, *** P < 0.001 vs. control, ### P < 0.001 vs. SDT.

MMPs are thought to be responsible for ECM turnover and degradation [20]. To investigate the potential mechanisms underlying the relative increase of collagen content upon DVDMS-SDT treatment in the rabbit and ApoE-/- mouse plaques, we investigated levels of multiple MMPs, including collagenases (MMP-1, 8), gelatinases (MMP-2, 9), matrilysin (MMP-7), and stromelysins (MMP-3, 10) using ELISA analysis. We found that MMP-2 and MMP-9 production was significantly lower in the THP-1-derived foam cells than in the control group at 5 h after DVDMS-SDT treatment (S3 Fig in S1 File). Consistent with the *in vitro* results, DVDMS-SDT substantially reduced the percentage of MMP-2 and MMP-9 by 51.3% (5.29 ± 2.41 vs. 2.57 ± 0.95%) and 49.7% (9.09 ± 2.90 vs. 4.58 ± 1.29%), respectively, in rabbit plaques (Fig 3A and 3B) 1 month after DVDMS-SDT treatment compared with the control group. Moreover, the percentages of MMP-2 and MMP-9 were drastically reduced by 57.5% (8.75 ± 2.31 vs. 3.71 ± 2.05%) and 61.5% (8.67 ± 2.13 vs. 3.34 ± 2.40%) in ApoE-/- mouse plaques (S4A, S4B Fig in S1 File). Additionally, the inhibitory effects of MMP-2 and MMP-9 production on DVDMS-SDT treatment in the THP-1-derived (Fig 3C and 3D) and MPM-derived (S4C, S4D Fig in S1 File) foam cells were efficiently blocked by pre-treatment with the ROS scavenger NAC or pan-caspase inhibitor Z-VAD-FMK.

### 3.3. DVDMS-SDT inhibits MMP-2 / MMP-9 expression via the caspase 3 –PEDF/HIF-1α signaling pathway in macrophage-derived foam cells

**Fig 4:**
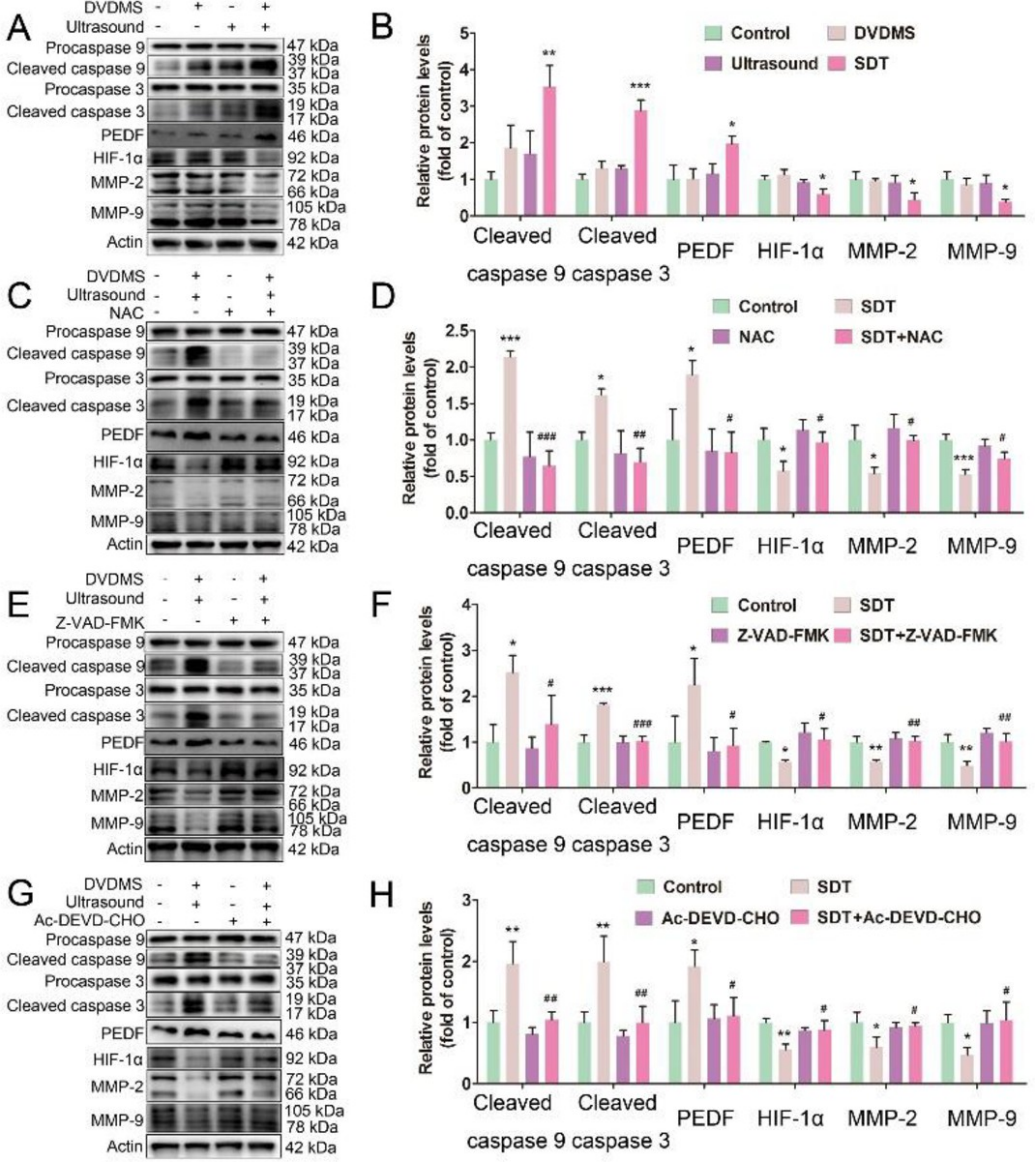
Sinoporphyrin sodium-mediated sonodynamic therapy (DVDMS-SDT) increases the protein level of PEDF and reduces the protein levels of HIF-1α / MMP-2 / MMP-9 via mitochondria-caspase pathway in THP-1-derived foam cells. THP-1-derived foam cells were pre-treated with NAC, Z-VAD-FMK or Ac-DEVD-CHO for 1 h, followed by DVDMS-SDT treatment. At 6 h after treatment, the cells in the indicated groups were collected for western blot. **(A, C, E, G)** Immunoblotting of caspase 9, caspase 3, PEDF, HIF-1α, MMP-2, and MMP-9 proteins levels, and **(B, D, F, H)** respective quantification. * P < 0.05, ** P < 0.01, *** P < 0.001 vs. control. # P < 0.05, ## P < 0.01, ### P < 0.001 vs. SDT.

**Fig 5:**
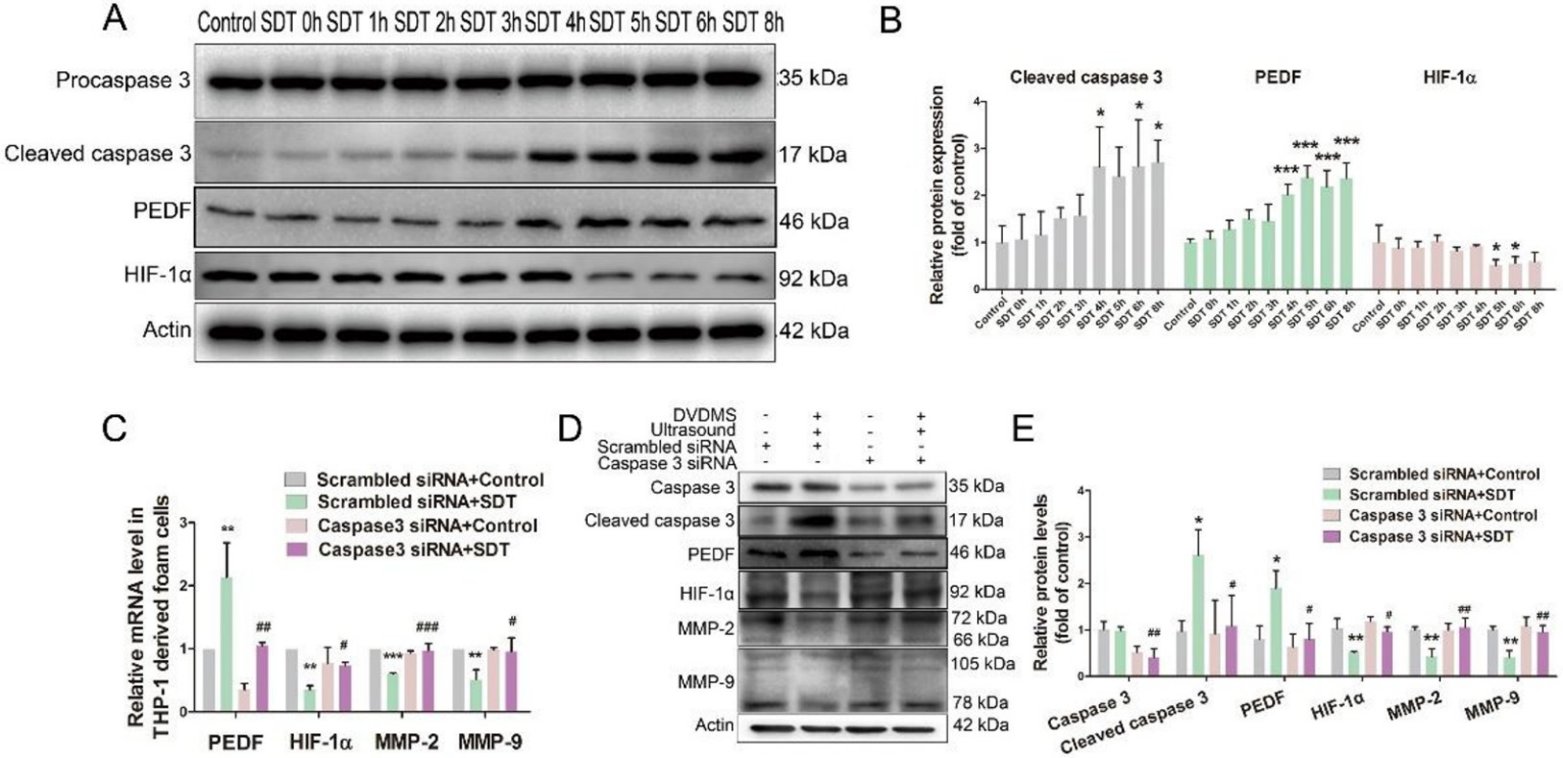
Sinoporphyrin sodium-mediated sonodynamic therapy (DVDMS-SDT) increases PEDF and inhibits HIF-1α/MMP-2/MMP-9 expression upon activation of caspase 3 in THP-1-derived foam cells. **(A)** Immunoblotting analysis of caspase 3, PEDF and HIF-1α proteins level and **(B)** respective quantification. **(C)** Quantitative real-time polymerase chain reaction analysis of the mRNA expression of PEDF, HIF-1α, MMP-2, and MMP-9 (n = 3). **(D)** Immunoblotting analysis of caspase 3, PEDF, HIF-1α, MMP-2, and MMP-9 proteins level and **(E)** respective quantification. * P < 0.05, ** P < 0.01, *** P < 0.001 vs. control or scrambled siRNA + control. # P < 0.05, ## P < 0.01, ### P < 0.001 vs. scrambled siRNA + SDT.

As in our previous study [16], we confirmed that DVDMS-SDT induced the mitochondrial caspase-dependent apoptosis in macrophage-derived foam cells (S5 Fig in S1 File). However, the role of caspase 3 in the regulation of MMP-2 and MMP-9 has yet to be documented. In our previous study, RNA sequencing and bioinformatics analysis showed that DVDMS-SDT downregulates a number of genes including SERPINF1 (the mRNA encoding pigment epithelium-derived factor [PEDF]) and HIF-1A, which was blocked by caspase 3 specific inhibitor Ac-DEVD-CHO [18]. Previous studies have indicated that PEDF inhibits the expression and activities of MMP-2/9 in the aqueous humor of a proliferative diabetic retinopathy model [21] and in spontaneous pancreatic carcinoma [22]. However, the transcription factor HIF-1α promoted MMP-2/9 expression [23, 24]. In this study, western immunoblotting analysis revealed that DVDMS-SDT significantly enhanced the protein expression of cleaved caspase 9, cleaved caspase 3, and PEDF, while reducing the protein expression of HIF-1α, MMP-2, and MMP-9 in macrophage-derived foam cells. These effects were efficiently nullified by the pre-treatment with NAC, pan-caspase inhibitor Z-VAD-FMK, or caspase 3-specific inhibitor Ac-DEVD-CHO (Fig 4). Additionally, we further confirmed that DVDMS-SDT increased PEDF and reduced HIF-1α, MMP-2, and MMP-9 mRNA and protein levels by activating caspase 3 (Fig 5). One day after DVDMS-SDT treatment in ApoE-/- mice, the number of cleaved caspase 3-positive and PEDF-positive macrophages in the plaque increased approximately 1.72-fold (70.75 ± 21.53 vs. 193.0 ± 35.42) (S6A, S6B Fig in S1 File) and 0.59-fold (68.0 ± 29.20 vs. 108.0 ± 23.56) (S6C, S6D Fig in S1 File), respectively, while the number of HIF-1α-positive macrophages decreased by approximately 50.8% (104.3 ± 30.22 vs. 51.33 ± 30.61) (S6E, S6F Fig in S1 File) compared with the control group.

## 4. Discussion

In the present study, we reveal the mechanistic pathway by which SDT inhibits matrix collagen degradation. Collagen is a principal constituent of atherosclerotic plaque that maintains plaque stability, and is primarily synthesized by vascular SMCs in the plaque [25]. Herein, we found that the percentage of collagen in rabbit and mouse advanced atherosclerotic plaques were significantly increased after DVDMS-SDT treatment, although the SMC area in the plaque remained unaltered. As MMPs produced by macrophages can degrade matrix collagen fragments in the atherosclerotic plaque [26], we found that DVDMS-SDT can significantly reduce the expression and secretion of MMP-2 and MMP-9 in plaque macrophages by measuring the secretion of a variety of MMPs, which is consistent with the findings of our previous study [15]. Therefore, we attributed the increase in the percentage content of collagen after the DVDMS-SDT treatment in this study to an inhibition of its degradation rather than an increase of its synthesis.

Recently, we found that DVDMS-SDT, as a macrophage-specific targeted treatment, activates caspase 3 via the mitochondrial-apoptosis pathway to inhibit the expression of SERPINF1 (the mRNA encoding PEDF) and HIF-1A [18], which are involved in the regulation of MMP-2 and MMP-9 [21–24]. In the present study, we found that DVDMS-SDT increased the expression of PEDF and decreased the expression of HIF-1 α by activating caspase 3 in macrophages, thereby inhibiting the expression of MMP-2 and MMP-9. The small sample size of RNA sequencing may explain why the PEDF results in this study contradict our RNA sequencing results.

Although HIF-1α protein is degraded post-translationally [27, 28], this process is disrupted by hypoxia, thus allowing HIF-1α to re-enter the nucleus and exert its transcriptional activity on target genes [28]. We previously found that DVDMS-SDT treatment cleaves SP-1 to inhibit HIF-1α transcription in macrophage foam cells [18]; however, the potential effect of the treatment on the degradation pathway of HIF-1α remains unknown. Our results achieved by western immunoblotting showed no remarkable variation in the expression level of HIF-1α protein during the first 4 h following DVDMS-SDT treatment, suggesting that the mode of action may have no effect on the HIF-1α degradation pathway.

A previous study showed that retinal glial cells upregulate PEDF production in an HIF-1α- and VEGF-dependent manner. In turn, PEDF inhibits the expression of HIF-1α, forming a feedback mechanism to suppress the overexpression of VEGF [29]. Moreover, PEDF can inhibit tumor angiogenesis by decreasing the expression of HIF-1α and VEGF [30]. These studies indicated that HIF-1α and PEDF are mutually regulated. In this study, we found that the expression of PEDF significantly increased due to activated caspase 3, while HIF-1α expression remained stationary, 4 h after DVDMS-SDT treatment. After 5 h of DVDMS-SDT treatment, the expression of HIF-1α had decreased significantly. These results suggested that caspase 3 activation by DVDMS-SDT first increases PEDF expression and then inhibits HIF-1α expression under the joint action of caspase 3 and PEDF.

## 5. Conclusions

**Fig 6:**
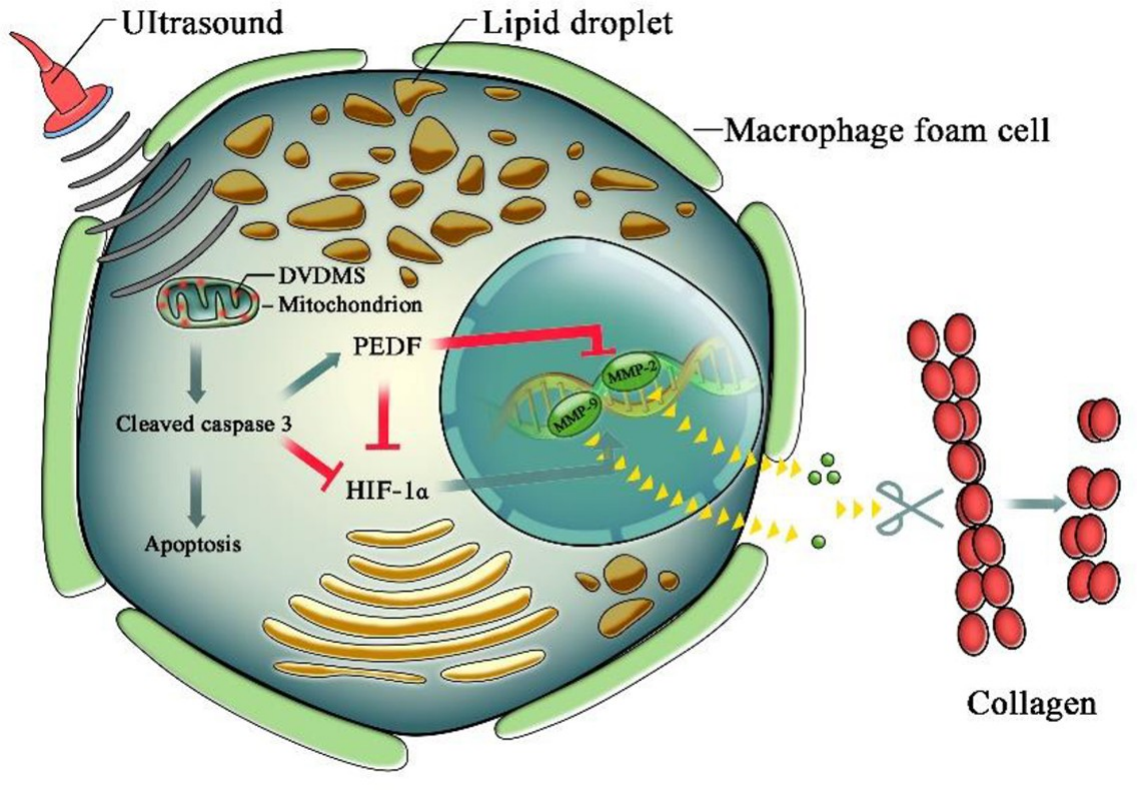
Illustration of sinoporphyrin sodium-mediated sonodynamic therapy (DVDMS-SDT) inhibition of collagen degradation in advanced atherosclerotic plaque. DVDMS-SDT induces mitochondrial-caspase apoptosis in macrophage foam cells. Meanwhile, activated caspase 3 increases PEDF expression, and caspase 3 and PEDF jointly inhibit HIF-1α expression, thereby suppressing the expression of MMP-2 and MMP-9 and resulting in the inhibition of collagen degradation.

As illustrated in Fig 6, DVDMS-SDT inhibits matrix collagen degradation in advanced atherosclerotic plaque by modulating caspase 3-PEDF/HIF-1α-MMP-2/MMP-9 signaling in macrophage foam cells. This non-invasive and local intervention may represent a promising procedure to stabilize vulnerable plaques in clinical applications.

## Supporting information

S1 File (DOCX)
