# Supplementary material for: Sonodynamic therapy suppresses matrix collagen degradation in vulnerable atherosclerotic plaque by modulating caspase 3 - PEDF/HIF-1α - MMP-2/MMP-9 signaling in macrophages
Source: PLoS One. 2022 Dec 27;17(12):e0279191. doi: 10.1371/journal.pone.0279191 (PMC9794047; doi:10.1371/journal.pone.0279191)
Supplement: S1 File — (DOCX) [file pone.0279191.s001.docx]

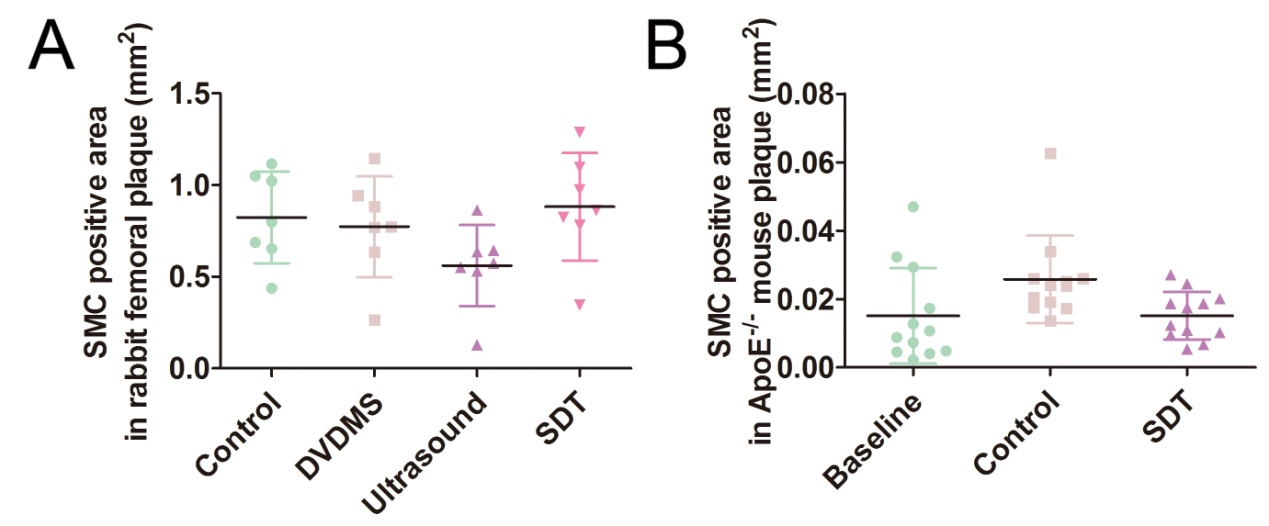


**Figure S1. The effect of DVDMS-SDT on the smooth muscle cell (SMC) positive area in rabbit femoral plaque and ApoE^-/-^ mouse plaque.** DVDMS-SDT had no effect on the smooth muscle cell (SMC) positive area in rabbit femoral plaque **(A)** and ApoE^-/-^ mouse plaque **(B)** at 1 month after treatment. (n = 7).


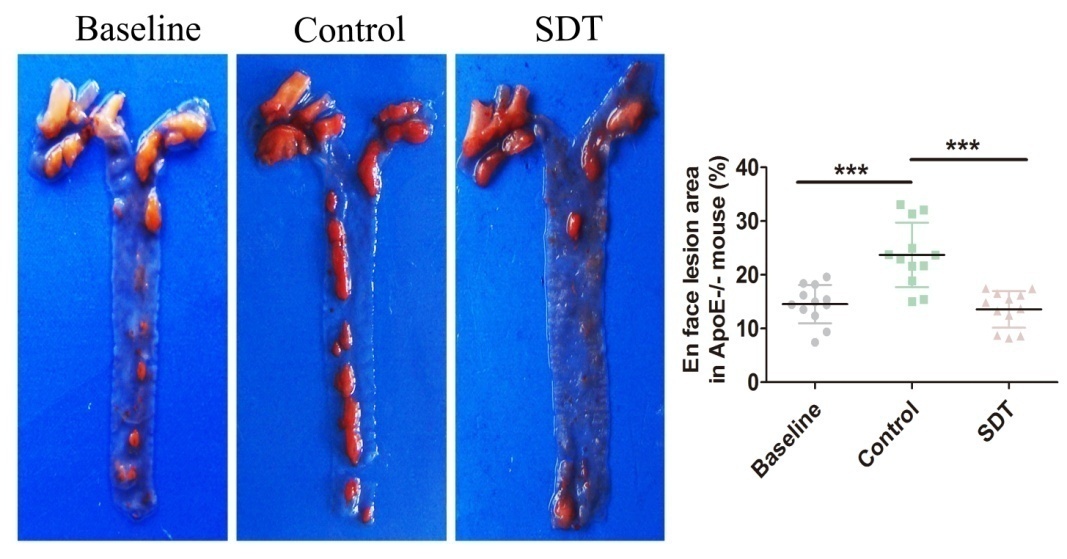


**Figure S2. DVDMS-SDT inhibited the progression of atherosclerotic plaque in ApoE^-/-^ mouse** **at 1 month after treatment.** DVDMS-SDT inhibited the progression of atherosclerotic plaque in ApoE^-/-^ mouse, as indicated by oil red O staining of aortas (n = 12). *** *P* < 0.001.


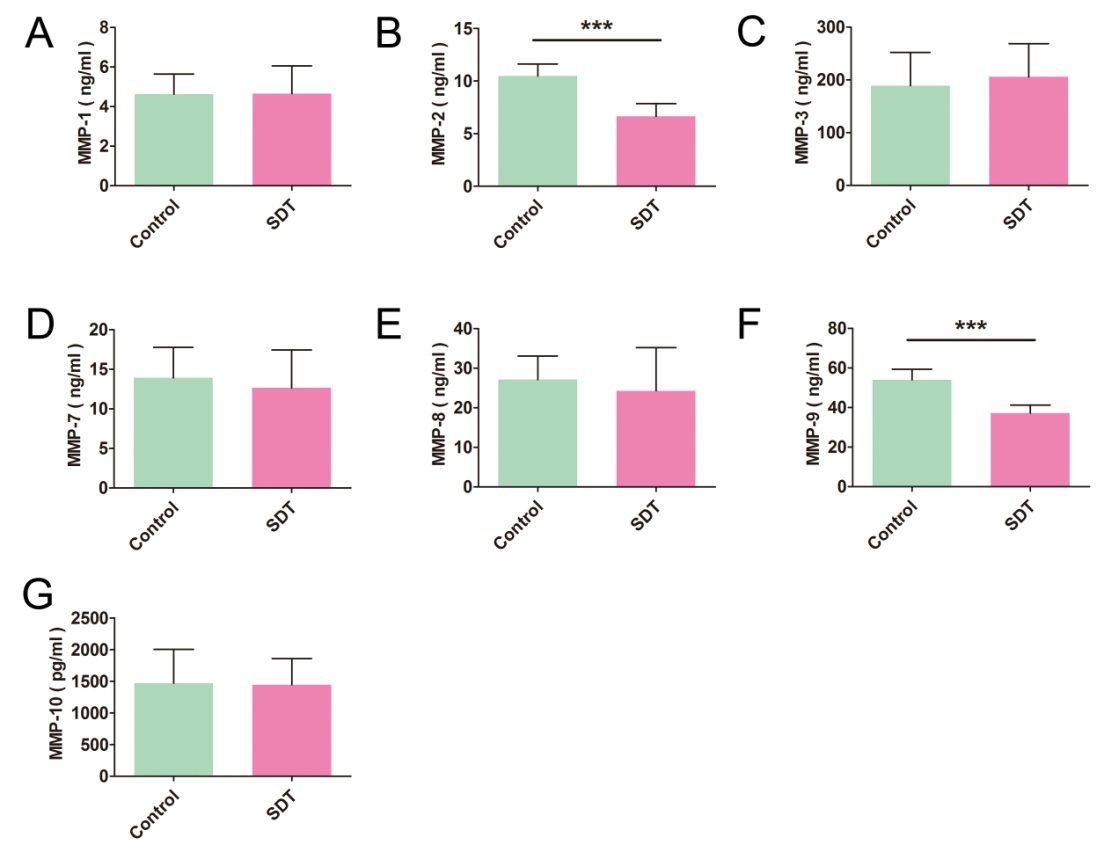


**Figure S3. DVDMS-SDT reduces MMP-2 and MMP-9 secretion in THP-1-derived foam cells.** At 5 h after DVDMS-SDT treatment, the supernatants of the THP-1-derived foam cells in the indicated groups were collected for determination of the concentration of MMP-1 **(A)**, MMP-2 **(B)**, MMP-3 **(C)**，MMP-7 **(D)**，MMP-8 **(E)**，MMP-9 **(F)** and MMP-10 **(G)** by ELISA. Data on graph were representative of three independent experiments. *** *P* < 0.001.

**
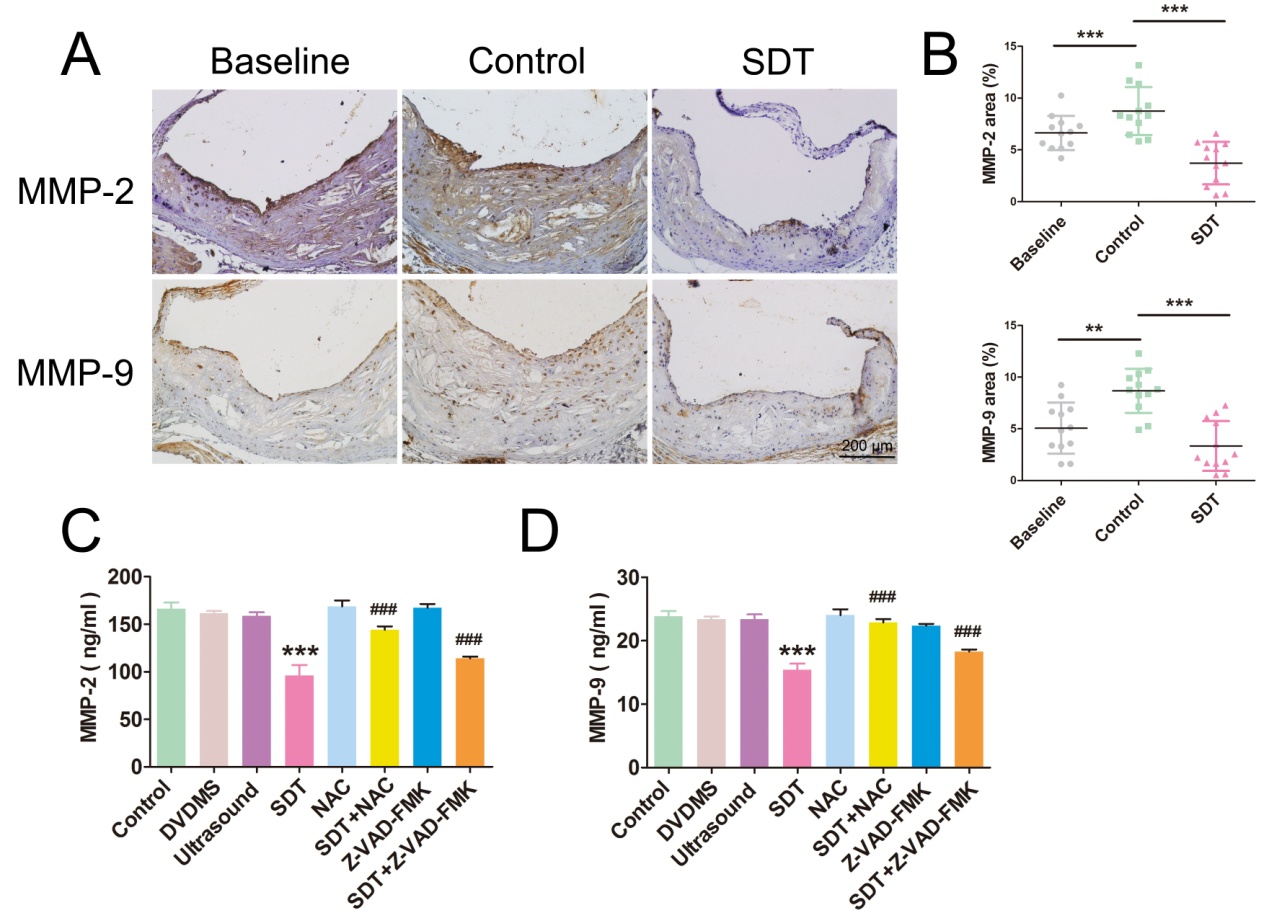
**

**Figure S4. DVDMS-SDT reduces the production of MMP-2 and MMP-9 in ApoE^-/-^ mouse advanced atherosclerotic plaque.** DVDMS-SDT reduced the expression of MMP-2 and MMP-9 in the ApoE^-/-^ mouse plaque at one month after treatment. Representative histopathological staining of consecutive plaque sections **(A)** and quantification **(B)** (n = 12). Mouse peritoneal macrophage (MPM)-derived foam cells were pre-treated with NAC or Z-VAD-FMK for 1 h, followed by DVDMS-SDT treatment. At 6 h after treatment, the supernatants in the indicated groups were collected for determination of the concentration of MMP-2 **(C)** and MMP-9 **(D)** by ELISA. Data on graph were representative of three independent experiments (n = 8). NAC = N-acetyl-L-cysteine. Z-VAD-FMK = methyl (3S)-5-fluoro-3-[[(2S)-2-[[(2S)-3-methyl-2 (phenylmethoxycarbonlamino)butanoyl]amino] propanoyl]amino]-4-oxopentanoate. ** *P* < 0.01, *** *P* < 0.001 *vs.* control, ^###^ *P* < 0.001 *vs.* SDT.


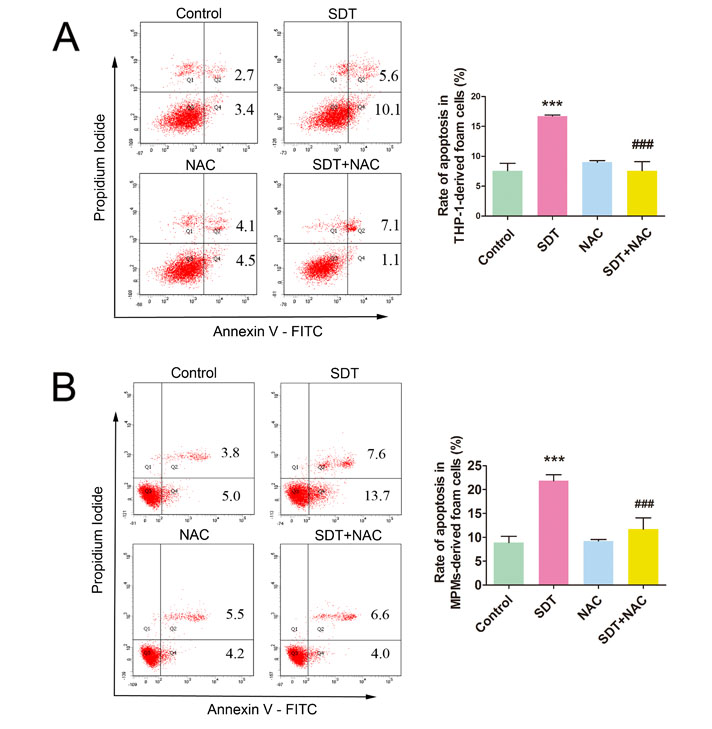


**Figure S5. DVDMS-SDT induces apoptosis in THP-1-derived (A) and MPM-derived (B) foam cells.** Cells were pre-treated with 10 mM NAC for 1 h, followed by DVDMS-SDT treatment. Six hours after treatment, the cells were harvested and stained with Annexin V-FITC/PI to analyze apoptotic cell populations by flow cytometry. Data on graph were from three independent experiments. *** *P* < 0.001 *vs.* control. ^###^ *P* < 0.001 *vs.* SDT.


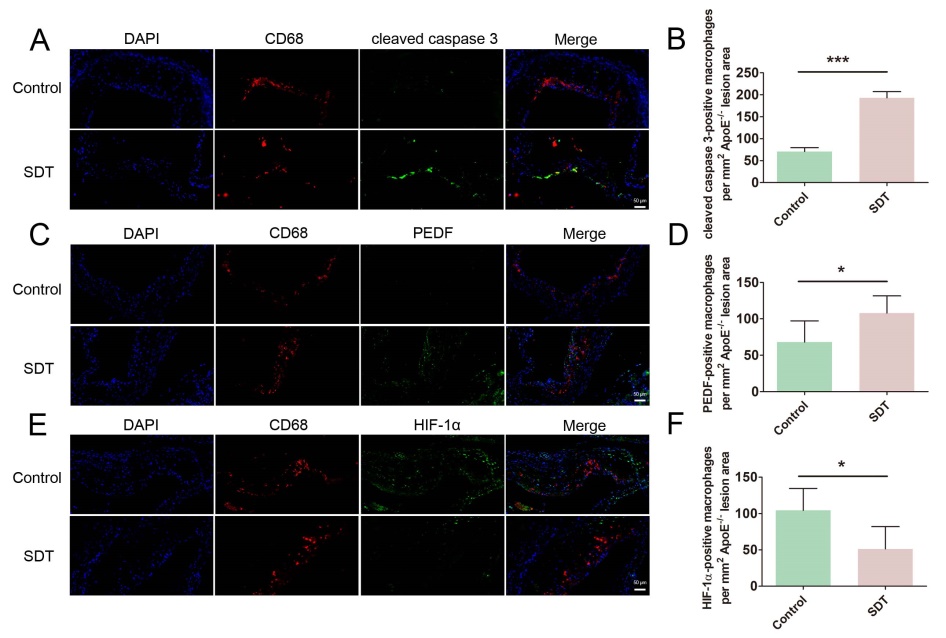


**Figure S6. The effect of DVDMS-SDT on the number of cleaved caspase 3-positive, PEDF-positive and HIF-1α-positive macrophages in ApoE^-/-^ mouse plaque at day 1 after treatment.** Representative aortic sections **(A)** and quantification of cleaved caspase 3-positive macrophages in plaque **(B)**. Representative aortic sections **(C)** and quantification of PEDF-positive macrophages in plaque **(D)**. Representative aortic sections **(E)** and quantification of HIF-1α-positive macrophages in plaque **(F)**. * *P* < 0.05, *** *P* < 0.001.
